# Measuring quality of life among people living with HIV: a systematic review of reviews

**DOI:** 10.1186/s12955-017-0778-6

**Published:** 2017-11-15

**Authors:** Vanessa Cooper, Jane Clatworthy, Richard Harding, Jennifer Whetham, Emerge Consortium, Adrian Brown, Agathe Leon, Eva Gonzalez, David Garcia, Daniel Lockhart, Ben Marent, Brian West, Cesar Caceres, Duncan Fatz, Ed Wallitt, Eduard Beck, Enrique Gomez, Eugenio Teofilo, Felipe Garcia, Flis Henwood, Goncalo Rodrigues, Jennifer Whetham, Josip Begovac, Koen Block, Ludwig Apers, Letitia Pereira, Margarida Borges, Mary Darking, Paloma Chausa, Sime Zekan, Steven Bremner, Steven Hoornaert, Sundhiya Mandalia

**Affiliations:** 1 The Lawson Unit, Brighton and Sussex University Hospitals NHS Trust, Eastern Road, Brighton, BN2 1HS UK; 2 Department of Palliative Care, Policy and Rehabilitation, King’s College London, Faculty of Life Sciences and Medicine, Cicely Saunders Institute, Bessemer Road, London, SE5 9PJ UK

**Keywords:** Quality of life, HIV, Systematic review, Validity, Reliability

## Abstract

**Aim:**

A systematic review of reviews was conducted to identify and appraise brief measures of health-related quality of life (HRQoL) that have been used in peer-reviewed research with people living with HIV.

**Methods:**

The review was conducted in two stages: 1) search of electronic databases to identify systematic reviews of tools used to measure HRQoL in adults living with HIV, published since the year 2000; 2) selection of HRQol scales from those identified in the reviews. Inclusion criteria included scales that could be self-administered in 10 min or less, covering at least 3 domains of quality of life (physical function, social/role function and mental/emotional function). For generic scales, inclusion criteria included the availability of normative data while for HIV-specific scales, patient input into the development of the scale was required.

**Results:**

Ten reviews met the inclusion criteria. Nine generic scales met the inclusion criteria: the EuroQol five dimensions questionnaire (EQ-5D); Health Utilities Index; McGill Quality of Life questionnaire; Medical Outcomes Study (MOS) Short Form (SF)-12; SF-36; World Health Organisation Quality of Life (WHOQOL- BREF), Questions of Life Satisfaction (FLZM) and SF-20. Available psychometric data supported the EQ-5D and SF-36. Seven HIV-specific scales met the inclusion criteria: the AIDS Clinical Trials Group (ACTG)-21; HIV-QL-31; MOS-HIV; Multidimensional Quality of Life Questionnaire for Persons with HIV/AIDS (MQOL-HIV), PROQOL-HIV, Symptom Quality of Life Adherence (HIV-SQUAD) and the WHOQOL-HIV BREF. Of the HIV -specific measures, the MOS-HIV was considered to have the most well-established psychometric properties, however limitations identified in the reviews included insufficient input from people living with HIV in the development of the scale, cross-cultural relevance and continued applicability. Two relatively new measures, the WHOQOL-HIV BREF and PROQOL-HIV, were considered to have promising psychometric properties and may have more relevance to people living with HIV.

**Conclusion:**

The findings highlight the need for further validation of HRQoL measures in people living with HIV. The choice of one measure over another is likely to be influenced by the purpose of the quality of life assessment and the domains of HRQoL that are most relevant to the specific research or clinical question.

## Background

Combination antiretroviral therapy (ART) has changed HIV from a terminal disease to a chronic condition in countries where treatment is widely available. With appropriate treatment, people with HIV can now have a near-normal life-expectancy [1]. However, people with HIV continue to have substantially lower health-related quality of life (HRQoL) than the general population, even where the majority of those living with HIV have virological control and are immunologically stable [2]. Evidence suggests that in addition to the underlying infection, social circumstances, relationship issues, comorbidities and stigma may impact on HRQoL in people with HIV [3].

HRQoL is a multidimensional construct concerned with the impact of health on an individual’s perception of their wellbeing and level of functioning in important areas of their life [4]. There is a lack of consensus regarding the specific dimensions of quality of life [5]. The constitution of the World Health Organisation, adopted in 1946, states that “Health is a state of complete physical, mental and social well-being” [6]. Reflecting this, health related quality of life is often conceptualised as having physical, mental and social domains [7]. Concepts such as independence, spirituality and environmental factors are also considered relevant [8].

Improving quality of life is central to the care and support of people with HIV [9]. Evaluations of new treatments and interventions to improve healthcare require the measurement of HRQoL as well as clinical endpoints (CD4 count, viral load, progression to AIDS). Valid, reliable and responsive tools are required to evaluate the impact of these interventions on HRQoL. To date there have been a number of reviews conducted to identify and assess measures of HRQoL in people with HIV, but these reviews have had diverse aims. For example, some have looked only at specific pre-selected measures (MOS-HIV and EQ-5D [10]) or measures that have been applied in a specific context (e.g. clinical trials [4, 11], developed countries [12], or sub-Saharan Africa [13]). The aim of this review is to identify brief, validated, pragmatic tools for appropriate assessment of HRQoL in HIV interventions and routine clinical care. It was not our intention to analyse the measurement properties of HRQoL tools, but to synthesise the findings of existing reviews.

## Method

The methodology was based on published recommendations for conducting systematic reviews of reviews [14]. As there was no specific guidance available for reporting on systematic reviews of reviews, the PRISMA guidelines [15] were used as a guide.

### Search strategy

Papers were identified in two ways:

1. Four online databases (Cochrane Database of Systematic Reviews, Database of Abstracts of Reviews of Effects, Medline, PsycINFO) were searched using the terms listed in Table 1.
2. Hand searching of reference lists of reviewed papers.

**Table 1:** Search Terms

HIV	Quality of Life	Measure^a^	Review
Human Immunodeficiency Virus	QoL	Inventor^a^	
	PROM	Scale^a^	
Antiretroviral^a^	Patient Reported Outcome	Questionnaire^a^	
		Self-report^a^	
		Assessment^a^	
		Survey^a^	
		Tool^a^	
		Indicator^a^	
		Instrument^a^	

Notes Terms within columns were combined with ‘OR’, terms between columns were then combined with ‘AND’

^a^Denotes truncation

The searches were conducted in February 2016. Searches were limited to studies published since year 2000. No language limits were set at this stage.

References of identified articles were exported to EndNote and deduplicated. The references and abstracts were then exported into an Excel spreadsheet for abstract review. All abstracts were reviewed independently by two researchers (JC, VC) to identify potentially relevant papers. Disagreement was resolved through discussion and adjudication by RH and JW. Full text copies of papers that appeared relevant were obtained and subjected to further scrutiny by the two reviewers.

### Inclusion and exclusion criteria

Papers were included in the review if they met the following criteria:

#### Inclusion criteria

Criterion 1: the paper reviewed tools that had been used to measure quality of life among adults living with HIV.

Criterion 2: the paper reported findings from a literature review that reported a systematic search strategy.

#### Exclusion criteria

Criterion 1: the review reported on HRQoL measures in children/adolescents.

Criterion 2: the review was published prior to 2000. We were interested in the assessment of quality of life of people living with HIV after the introduction of highly active antiretroviral therapy (HAART) in 1996. The year 2000 was selected to allow for the conduct and publication of both individual empirical articles and subsequent review papers capturing HRQoL in the HAART era.

Criterion 3: conference abstract

Criterion 4: focused on quality of life in relation to a specific comorbidity/ treatment side effects (e.g. lipodystrophy).

### Analysis

The search was recorded using the PRISMA flowchart [15]. Two researchers (JC, VC) independently extracted the following data from the papers, where available: the aim of the review, dates of the search, databases and other sources searched, search terms, language restrictions, the number of papers reviewed, the generic and HIV-specific HRQoL measures identified.

In order to assess the quality of the reviews, two reviewers (JC, VC) independently applied existing quality criteria [16] that assess: 1) research question 2) eligibility criteria 3) search strategy, 4) selection of papers, 5) quality assessment 6) presentation of data 7) publication bias 8) heterogeneity. Disagreement was resolved through discussion.

### Inclusion of HRQoL measures for the review

The second stage of the review involved the selection of appropriate HRQoL measures from those identified in the reviews. A similar approach to that taken by Clayson et al. (2006) [4] was adopted, in order to identify comprehensive yet pragmatic tools for assessing HRQoL in HIV interventions and clinical care. Measures were deemed appropriate if they met the following criteria:

- Content: Coverage of at least three core domains of HRQoL: physical function, social/role function and mental/emotional health. In addition patient input was required in the development of HIV-specific measures, in order to reflect patients’ experience of disease.
- Practicality: Measures needed to be self-administered and typically completed within 10 min. Where completion time data were not available, we included measures with less than 40 items. This cut-off was based on an estimation of completion rates drawing on the data available in the reviews, indicating approximately four items per minute.
- Normative data: For generic measures, normative data needed to be available to allow comparison between people with HIV and the general population.

Information on each of the selected measures was then obtained from the reviews. This included the HRQoL domains addressed, number of items, type of scale, accessibility (e.g. availability in different languages, availability of population preference rates for generic measures, approximate time taken to complete the measure), reliability, validity (including responsiveness) and conclusions/recommendations made within the reviews.

## Results

**Fig. 1:**
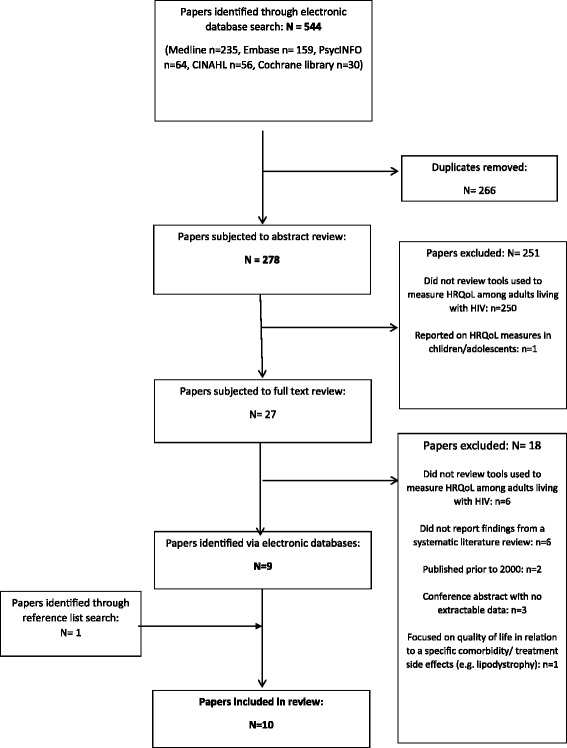
PRISMA Flow diagram showing paper selection process

Figure 1 shows the PRISMA flowchart. The electronic database search identified 544 papers. After removal of duplicates, 278 abstracts were subjected to review. After applying our inclusion and exclusion criteria, 27 articles were obtained and subjected to full text review. Of those, 9 met the inclusion criteria. One further study was identified through the reference list search.

### Overview of the published systematic reviews

Characteristics of the reviews are summarised in Table 2. The search periods for the published systematic reviews addressed the period 1995–2013 (6 studies did not record the dates of the search and one study did not record the start date). Four reviews were restricted to abstracts/papers published in the English language [3, 10–12], one was restricted to papers published in English or Spanish [17], one had no language restrictions [18] and the remaining 4 reviews did not state whether or not the review was restricted by language [4, 13, 19, 20]. The number of articles reviewed ranged from 26 to 852 (six did not specify the number of articles reviewed). The number of generic HRQoL measures identified by the reviews ranged from 0 to 23 (median = 7) and the number of HIV specific measures identified ranged from 1 to 18 (median = 11).

**Table 2:** Summary of reviews identified

Paper	Aim of the review	Dates of search	Databases and other sources	Search terms	Language restriction?	No. papers reviewed	No. HRQoL measures identified	No. generic HRQoL measures	No. HIV-specific measures
Carabin et al. (2008) [17]	To assist clinicians in choosing the most appropriate instrument to measure health related quality of life among people living with HIV.	Not stated	OVID, Pubmed and ERIC English language	Not stated	English and Spanish	Not stated	17	6	11
Clayson et al. (2006) [4]	To review existing HR-QOL measures reported in the HIV/AIDS literature since 1990 and identify those most worthy of consideration for use in clinical trials.	1990-not stated	General abstract databases such as MEDLINE, PsycINFO, INSPEC, EMBASE, CINAHL PRO instrument databases such as the On-Line Guide to Quality of Life Assessment (OLGA) and the Quality of Life Instruments Database (QOLID)	Not stated	Not stated	Not stated	34	17	17
			Cochrane Library Database International Society for Quality of Life Research and the International Society for Pharmacoeconomics and Outcomes Research abstracts and conference proceedings Specific HIV-related journals, including AIDS, AIDS Care, International Journal of STD & AIDS and Journal of Acquired Immune Deficiency Syndrome.						
Colautti et al. (2006) [18]	To review validated questionnaires to assess health related quality of life in HIV/AIDS patients	Not stated	PubMed, Medline and Medscape	“HRQL”, “questionnaire” and “HIV”	None	Not stated	20	9	11
Davis & Pathak (2001) [19]	To provide a comparative evaluation of psychometric properties of four HIV disease–specific quality-of-life (QoL) instruments.	Not stated	MEDLINE, Health Star, International Pharmacy Abstracts, Social Sciences Citation Index, Current Contents Manual reference list search	Not stated	Not stated	Not stated	4	0	4
Drewes et al. (2013) [3]	To provide a comprehensive summary of the methodological approaches used to study the QOL of people with HIV and AIDS based on published research.	To early 2011	PubMed and PsycINFO	“HIV/AIDS,” “quality of life,” and related concepts, like “well-being,” “life satisfaction,” and the names of established QOL instruments.	English abstract	852	>40 (but only report on the 14 most commonly used QOL measures)	8	6
Gakhar et al. (2013) [12]	To provide an overview of the Health Related Quality of Life tools available, the methods used for validation, the impact that ART has had on the HRQOL of HIV-infected people, as well as looking at some of the most important adverse effects of ARVs and co-morbidities.	Not stated	PubMed, Web of Science, Cochrane, MEDLINE and Scopus. Manual reference list search Internet databases to look for other HRQOL tools (http://www.proqolid.org).	Included “HIV,” “HRQOL Measures,” “quality of life” and “health status.”	English	Not stated	41	23	18
Robberstad & Olsen (2010) [13]	To review the existing evidence on health related quality of life in HIV/AIDS patients in sub-Saharan Africa and consider how this information is used in the economic evaluation literature.	Not stated	PubMed, Embase and ISI Expert input	“HIV OR AIDS”, “Africa south of the Sahara” AND “health related quality of life”	Not stated	29	24	6	18 (only describe 3)
Simpson et al. (2013) [11]	To identify and classify PRO instruments used to measure treatment effects in clinical trials evaluating NNRTIs.	March 2003–Feb 2013	PubMed Manual reference list search	Included a combination of MeSH terms for HIV [HIV OR HIV infections], MeSH terms associated with PROs/instruments [questionnaires OR interviews as topic OR quality of life OR patient satisfaction OR self-evaluation programs], Substance Names of NNRTIs [efavirenz OR nevirapine OR delavirdine OR etravirine OR rilpivirine OR efavirenz, emtricitabine, tenofovir disoproxil fumarate drug combination], and clinical trial Publication Types [clinical trial OR clinical trial, phase IV OR clinical trial, phase III OR clinical trial, phase II OR controlled clinical trial OR randomized controlled trial]	English	26	8	4	4
Skevington & O’Connell (2003) [20]	To address issues surrounding the measurement of quality of life of people living with HIV and AIDS and discuss the properties of suitable instruments	Jan 1995–May 2000	EMBASE, MEDLINE, the Web of Science (Version 4.1), PubMed. And Psychlit	‘quality of life’ with ‘HIV’ and/or ‘AIDS’ Names of particular QOL scales (e.g. Euroqol, SF-36) AND HIV/AIDS	Not stated	Not stated	21	10	11
Wu et al. (2013) [10]	To examine the responsiveness of two health-related quality of life (HRQL) instruments used in clinical trials involving HIV-infected adults: the HIV-targeted Medical Outcomes Study HIV Health Survey (MOS-HIV), and a generic measure, the EuroQol-5D (EQ-5D).	2001–2010	PubMed Manual reference list search	Included a combination of MeSH terms for HIV [HIV OR HIV infections], instrument names [Euroqol, EQ-5D, MOS-HIV], and clinical trial Publication Types [clinical trial OR clinical trial, phase IV OR clinical trial, phase III OR clinical trial, phase II OR controlled clinical trial OR randomized controlled trial]	English	17	2	1	1

### Quality of the reviews

The results of the quality assessment [16] are shown in Table 3. Eight of the ten reviews clearly stated a well formulated research question. Four of the ten reviews had predefined and specified their inclusion and exclusion criteria, and 4 had conducted a comprehensive search including multiple scientific literature databases and manual searches of references. None of the reviews stated that at least two researchers had conducted an independent review of titles, abstracts and full text review and indicated how disagreements between the reviewers were resolved. No reviews included a quality assessment. Two reviews listed the studies they included along with descriptions of their key characteristics. None of the reviews assessed publication bias. The criterion ‘was heterogeneity assessed’ [16] was not applicable because none of the reviews included a meta-analyses.

**Table 3:** Quality assessment applied to the identified reviews

Paper	1. Is the review based on a focused question that is adequately formulated and described?	2. Were eligibility criteria for included and excluded studies predefined and specified?	3. Did the literature search strategy use a comprehensive systematic approach?	4. Were titles, abstracts, and full-text articles dually and independently reviewed for inclusion and exclusion to minimize bias?	5. Was the quality of each included study rated independently by two or more reviewers using a standard method to appraise its internal validity?	6. Were the included studies listed along with important characteristics and results of each study?	7. Was publication bias assessed	8. Was heterogeneity assessed? (this question only applies to meta-analyses)
Carabin et al. (2008) [17]	N	N	N	NR	N	N	N	NA
Clayson et al. (2006) [4]	Y	N	NR	NR	N	N	N	NA
Colautti et al. (2006) [18]	Y	N	N	NR	N	N	N	NA
Davis & Pathak (2001) [19]	Y	N	NR	N	N	N	N	NA
Drewes et al. (2013) [3]	Y	Y	N	N	N	N	N	NA
Gakhar et al. (2013) [12]	Y	N	NR	NR	N	N	N	NA
Robberstad & Olsen (2010) [13]	Y	Y	N	NR	N	N	N	NA
Simpson et al. (2013) [11]	Y	Y	N	NR	N	Y	N	NA
Skevington & O’Connell (2003) [20]	N	N	N	NR	N	N	N	NA
Wu et al. (2013) [10]	Y	Y	N	NR	N	Y	N	NA

Y yes; N No; NA not applicable; NR not reporte**d**

### Selection of measures for further scrutiny

#### Generic measures

**Fig. 2:**
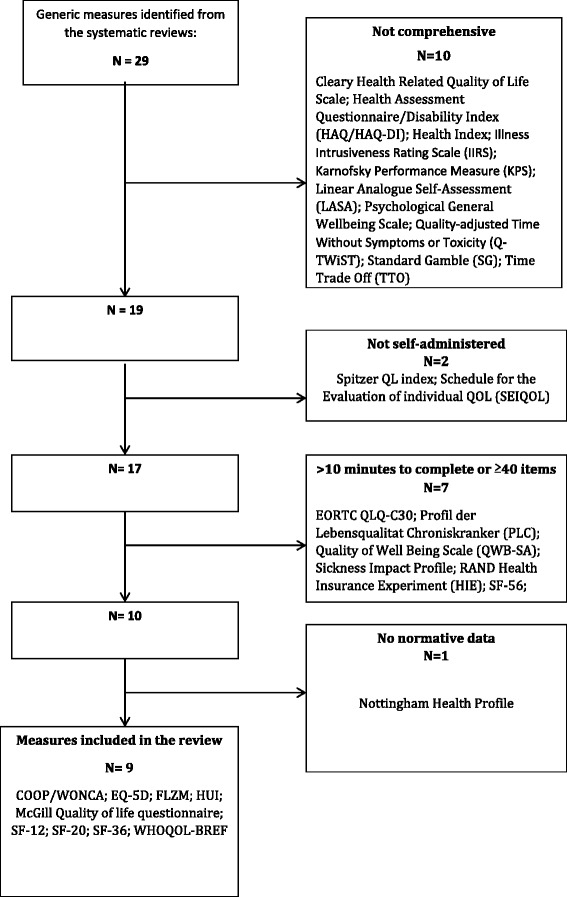
Flow diagram showing selection of generic measures

Twenty-nine generic HRQoL measures were reviewed against the inclusion criteria (Fig. 2). Of those, 10 were not comprehensive, 2 were not self-administered, 7 took longer than 10 min to complete/ had 40 or more items and 1 had no normative data and were therefore excluded. Nine generic measures met the inclusion criteria and were retained for further analysis. These were the COOP/WONCA charts [21], EQ-5D [22, 23], FLZM Questions on Life Satisfaction [24], HUI [25], McGill Quality of life questionnaire [26], SF-12 [27], SF-20 [28], SF-36 [29–31] and WHOQOL-BREF [32, 33].

#### HIV-specific measures

**Fig. 3:**
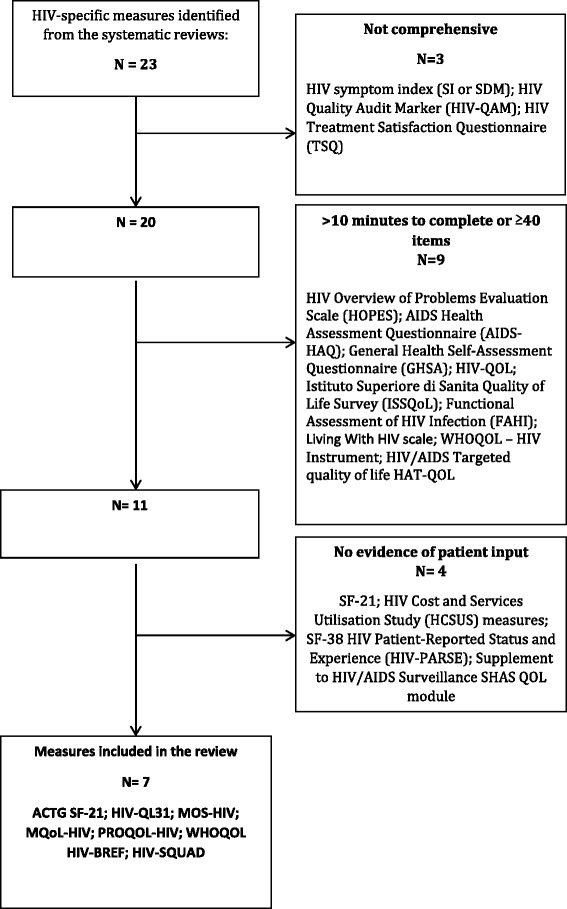
Flow diagram showing selection of HIV-specific measures

Twenty-three HIV-specific HRQoL measures were reviewed against the inclusion criteria (Fig. 3). Of those, 3 were not comprehensive, 9 took longer than 10 min to complete/ had 40 or more items and there was no evidence of patient input in the development of 4 of the measures. Seven HIV-specific measures therefore met the inclusion criteria and were retained for further analysis. These were the ACTG SF-21 [34], HIV-QL31 [35], MOS-HIV [36], MQoL-HIV [37], PROQOL-HIV [38], WHOQOL HIV-BREF [39] and HIV-SQUAD [40].

### Overview of selected generic HRQoL measures

Properties of each of the generic HRQoL measures are summarised in Table 4.

**Table 4:** Qualities and psychometric properties of the selected generic measures extracted from the reviews

Scale	Domains addressed	Completion time/Number of items	Response format	Accessibility	Validity	Reliability	Responsiveness	Floor/ Ceiling effects	Conclusions/recommendations of the reviews
COOP/WONCA [21]	Physical fitness, feelings, daily activities, social activities, pain, change in health, overall health, social support and quality of life	<5 mins [58] 9 items (more recently reduced to 6)	Five options with pictorial depictions accompanying the text	Available in 20 languages [18] Acceptable and feasible [13]	HIV positive women had poorer scores than HIV negative women on six out of nine health dimensions (construct validity) [13]	not stated	not stated	not stated	not stated
EQ-5D [22, 23]	Mobility; self-care, usual activities, pain/discomfort, anxiety/depression, self-reported health	1 min [4] 6 items	5 dimensions of quality of life are rated on either a 3 point scale (no problems/ some or moderate problems/ extreme problems – EQ-5D-3 L) or 5 point scale (no problems/ slight problems/ moderate problems/ severe problems/ extreme problems – EQ-5D-5 L). In addition, a visual analogue scale (0–100) is used to rate overall health.	Approximately 1 min to complete [4]. Available in multiple languages [12, 18]. Can be administered electronically or over phone [12]. General population preference weights have been derived for many countries [4].	Correlates with MOS-HIV subscales and discriminates between participants stratified by HIV/AIDS severity based on CD4 count/viral load (construct validity) [4, 12]. Lower EQ-5D scores among people with HIV not receiving ART than general population (construct validity) [13].	not stated	Responsive to initiation of ART, the development of opportunistic infections and adverse events [4, 10, 12, 13], with small-to-medium effect sizes in each of its five dimensions [10].	Ceiling effects in general population samples [4, 12, 20].	Clayson et al. recommend using the EQ-5D alongside a disease-targeted measure, however because of ceiling effects in general population samples they would not recommend the EQ-5D for studies including individuals with early, asymptomatic HIV infection. [4] Wu et al. recommend use alongside the MOS-HIV to obtain HIV-specific HRQL and utility measures [10]. Performance equivalent to the MOS-HIV in clinical trials [20]. Can generate indirect health utility values for use in economic models [10, 11].
FLZM Questions on life satisfaction [24]	Satisfaction with life in general: friends’ free time, general health, financial security, work, life conditions, family life and relationships. Satisfaction with health: physical condition, ability to rest, energy, mobility, freedom from anxiety, freedom from pain, independence	“A few minutes” [24] 16 items	5 point scales rating the importance of and satisfaction with each aspect of quality of life.	not stated	not stated	not stated	not stated	not stated	not stated
Health Utilities Index (HUI) HUI2; HU13 [25]	Vision, ambulation, dexterity, emotion, cognition, hearing, speech and pain	5–10 mins [59] 15 items	4–6 response options for each question	Available in multiple languages [12]. Can be administered electronically [12].	HUI2 and 3 have been associated with disease severity/AIDS related events and plasma viral load (construct validity). [4] Correlates well with most MOS-HIV subscales (convergent validity) [4, 12, 20].	not stated	Responsive to change in HIV disease states [4, 12, 20], however the MOS-HIV and the EQ-5D VAS had better discriminatory capacity [12].	not stated	Despite less evidence for the HUI than EQ-5D and SF-36, emerging data were positive. [4] Potentially useful adjuvant to an HIV-specific measure in a trial. [4]
McGill Quality of life questionnaire (M-QOL) [26]	Physical, Psychological, Existential, Support.	16 items	Two response options for each item (e.g. no problem vs tremendous problem)	not stated	Content/face validity: The existential dimension is particularly relevant to people with advanced disease (CD4 < 100) [20] Only scores for physical symptoms distinguished between people with HIV with low and high CD4 count (construct validity). [20]	Factor analysis indicated four reliable subscales plus a single item about physical wellbeing (internal consistency). [20]	not stated	not stated	not stated
SF-12 [27]	Physical functioning, role physical, bodily pain, general health, vitality, social functioning, role emotional, mental health	2–3 mins [60] 12 items	2–6 response options per item	not stated	not stated	No internal consistency data reported [17].	Mixed results in terms of responsiveness to change in treatment [12].	Likely to have similar floor and ceiling effects to other MOS measures [17].	Clayson et al. recommend the use of the SF-12 where the length of the SF-36 is a problem. [4]
SF-20 [28]	not stated	3–5 mins [61] 20 items	3–6 response options per item	not stated	No construct validity data [17].	Adequate cronbach’s alphas (internal consistency) [17].	not stated	Floor and ceiling effects noted in some dimensions [17].	not stated
SF-36 [29–31]	Physical functioning, role-physical, bodily pain, general health, vitality, social functioning, role-emotional, mental health, reported health transition	7–10 mins [4] 36	2–6 response options per item	Has been translated into several different languages [18, 20]. Dominates generic HR-QOL measurement with normative scores for US, UK and many other countries [4, 20]. Takes 7–10 min to complete [4]. Can be administered electronically [18]	Correlates with disease severity, CD4 counts and other measures of QOL.(construct validity) [12] PLWL reported lower QOL on all dimensions, compared to healthy controls [12, 13, 20], with the biggest decline between Stages 1 and 2 of the disease (construct validity) [13, 20]. Scale scores were associated with treatment duration, less co-morbidity, and better social support improved physical functioning (construct validity) [13].	Cronbach’s alpha within acceptable range (internal consistency) [12, 17].	Responsive to the initiation of ART and change in CD4 count, viral load and the number of symptoms. [4] Improvement in all HRQOL domains along with clinical indicators after starting ART. May not be sensitive to change of ART medication in people with HIV who are stable on ART [12].	Problems with floor and/or ceiling effects for some subscales [17, 20].	More evidence for the SF-36 in people with HIV than other recommended generic measures (EQ-5D or HUI) and the SF-12 is a viable alternative if the length is a problem. [4] Coluatti et al. recommend the SF-36 as the most appropriate generic measure for assessing HRQL in people with HIV [18]. Use alongside a disease-targeted measure (other than the MOS-HIV which shares items) is recommended. [4] Unclear whether there is an advantage to using the MOS-HIV over the SF-36. [4] This and other MOS measures were developed in US – although translated into other languages people from these countries had no input into development and these versions may have limited semantic equivalence [20]. Can be used in cost-utility analyses by deriving utility weights from the SF-36 [11].
WHOQOL-BREF [32, 33]	Physical health, psychological health, social relationships and environment.	<5 mins [20] 26 items	5-point scales	Available in 40 languages. Takes <5 min to complete [20]. Developed in 15 centres worldwide to increase cross-cultural validity [12].	Correlates well with disease severity, patients who had lower CD4 counts had lower HRQOL (construct validity) [12].	Cronbach’s alpha coefficients in the acceptable range (internal consistency) [12].	not stated	not stated	Developed from the WHOQOL-100 measure, which was developed within an international collaboration of 15 countries using a spoke-wheel methodology to ensure conceptual and semantic equivalence [20].

### *COOP/WONCA* [21]

The COOP/WONCA charts contain 6–9 items including the following domains: physical fitness, social activities, feelings, change in health, daily activities and overall health. Each item has five response options, presented pictorially. They are available in several languages and have been found to be acceptable to patients, however very little psychometric data were available in the reviews and they have not been widely used with people with HIV. Therefore, insufficient evidence is available to determine the suitability of this instrument for use in HIV research or clinical practice.

### *EQ-5D* [22, 23]

The EQ-5D consists of 5 items encompassing five dimensions of quality of life (mobility, self-care, usual activities, pain/discomfort, anxiety/depression) and an optional visual analogue scale to rate overall health. It takes approximately 1 min to complete and is available in multiple languages. General population preference weights have been derived for many countries, enabling the use of the measure in economic analyses. The reviews provided evidence of construct and convergent validity, as well as responsiveness to treatment initiation, the development of opportunistic infections and adverse effects with small to medium effect sizes [4, 10, 12, 13]. The measure has been frequently used in research with people with HIV, and several authors recommended it for use in this population [4, 10, 13]. However, problems with ceiling effects were noted [4, 20] therefore the use of the scale in individuals with early, asymptomatic HIV infection was not recommended [4, 19]. Wu et al. (2013) recommended the use of EQ-5D alongside an HIV-specific measure (the MOS-HIV) in order to obtain HIV-specific quality of life alongside this utility measure [10].

### Health utilities Index [25]

The Health Utilities Index is available in two versions (HUI mark 2 (HUI2) and HUI mark 3 (HUI3)), which consist of 15/16 items respectively and assess 7 domains – vision, ambulation, dexterity, emotion, cognition, hearing, speech and pain. The measures are available in multiple languages. Although the scales have not been extensively used in HIV research, there is some evidence for their validity in this context. HUI2 and HUI3 have been associated with disease severity, AIDS-related events and viral load. [4, 12] Construct validity has been established by correlating HUI scales with the MOS-HIV [4, 20]. The scales have also been found to be responsive to change in HIV disease states [12, 20] however, both the MOS-HIV and EQ-5D have been found to have better discriminatory capacity [12]. Based on emerging data, Clayson et al. (2006) considered the HUI to be a potentially useful adjuvant to an HIV specific measure [4].

### The McGill quality of life questionnaire (M-QOL) [26]

The McGill Quality of Life Questionnaire comprises 16 items encompassing four domains: support, existential well-being, physical and psychological symptoms. Skevington et al. report that the face/content validity of the measure is improved by the inclusion of an existential dimension, which may be particularly relevant to people with HIV who have advanced disease (CD4 count <100) [20]. However, only scores for one subscale (physical symptoms) distinguished between people with HIV who had high and low CD4 counts, indicating poor discriminant validity of the other subscales [20]. There was a lack of information in the reviews on responsiveness or reliability. This measure has not been frequently used with people with HIV. There was therefore insufficient information available to determine the suitability of this instrument.

### SF-12 [27]

The SF-12 is one of three generic measures (along with the SF-20 and SF-36) from the Medical Outcomes Study (MOS) that met our inclusion criteria. It consists of 12 items in 8 domains: physical functioning, role-physical, role-emotional, bodily pain, general health, vitality, social functioning and mental health, allowing for the generation of physical and mental health summary scores. There were little validity and reliability data available in the reviews and mixed results were presented in terms of responsiveness to change in treatment [12]. Since the SF-12 shares items with other measures developed from the MOS, it was considered likely that the scale had similar floor and ceiling effects to other MOS scales [17]. Clayson et al. recommended the use of the SF-12 in place of the SF-36 where the length of the SF-36 is a problem [4].

### SF-36 [29–31]

The SF-36 comprises 36 items encompassing 9 domains: physical functioning, role-physical, bodily pain, general health, vitality, social functioning, role-emotional, mental health and reported health transition allowing for the generation of physical and mental health summary scores. The scale has been translated into several languages and takes approximately 10 min to complete. Scores on the SF-36 can be used in economic analyses by deriving utility weights [11]. The original SF-36 [28] was modified to improve the range and precision of some of the scales, improve the wording and format of the questionnaire, resulting in the SF-36 v2 [41], however the reviews largely failed to distinguish between the two versions. As a result, this review refers only to the SF-36 and does not specify whether the findings relate to Version 1 or Version 2. The reviews reported good to acceptable internal consistency and construct validity [13, 20]. People with HIV reported lower HRQoL on all dimensions compared to general population controls [13, 20]. Scale scores have been associated with treatment duration, less comorbidity and better social support [13]. The SF-36 has been found to be responsive to the initiation of ART, change in CD4 count, viral load and symptoms [4, 12, 20] however it may not be sensitive to change in ART medication in patients who are stable on ART [12]. Problems with floor and/or ceiling effects have been reported on some subscales [20]. The SF-36 was recommended for use in people with HIV in two of the reviews [4, 18], however a criticism of this and other MOS measures was that they were developed in US and translated into other languages without the input of patients to ensure conceptual and semantic equivalence, and therefore may not be relevant for use in cross-sectional research [20].

### WHOQOL-BREF [32, 33]

The WHOQOL-BREF was developed from the WHOQOL-100 instrument, which was produced within an international collaboration of 15 countries, using a spoke-wheel methodology to ensure conceptual and semantic equivalence across languages and cultures [20]. The instrument has been frequently used in people with HIV. It consists of 26 items over 4 domains: physical health, psychological health, social relationships and environment. It is available in 40 languages and takes less than 5 min to complete [20]. Good psychometric properties, including Cronbach’s alpha coefficients in the acceptable range, correlations with disease severity and CD4 count were reported [12, 20], however data on responsiveness were not available in the reviews.

Two additional instruments, the FLZM [24] and the SF-20 [28], were identified (Table 4), however the reviews did not assess their psychometric properties. There was insufficient information available to determine the suitability of these instruments.

### Overview of selected HIV specific HRQoL measures

Properties of each of the HIV-specific HRQoL scales are summarised in Table 5.

**Table 5:** Qualities and psychometric properties of the selected HIV-specific measures extracted from the reviews

Scale	Domains addressed	Completion time/No. items	Response format	Accessibility	Validity	Reliability	Responsiveness	Floor/ Ceiling effects	Conclusions/recommendations of the reviews
ACTG SF-21 [34]	Physical functioning, energy/fatigue, social functioning, role functioning, cognition, pain, health perception and emotional well-being.	4–5 mins [34] 21 items	3–6 response options per item plus a visual analogue scale	Available in 2 languages [17].	No validity data available [17].	No internal consistency reported [17].	not stated	Likely to have similar floor and ceiling effects to other MOS measures [17].	not stated
HIV-QL31 [35]	Sex life, pain, psychological aspects, relationships, limitations caused by HIV, symptoms, impact of treatment and care	31 items	Yes/No	Available in English and French [18].	Discriminates between groups with different severity levels (construct validity) [20].	High internal consistency [17, 20].	not stated	not stated	QL-31 is a relatively sound and useful instrument where attention has been paid to the breadth of the concept as a result of listening to the concerns of patients. However it is culture-specific in the ways it has been designed [20].
MQOL-HIV [37]	Mental health, physical health, physical functioning, social functioning, social support, cognitive functioning, financial status, partner intimacy, sexual functioning, perceived access to medical care	10 mins [4] 40 items	Likert scale (never – always)	In a study comparing the MQOL-HIV with the MOS-HIV there were more missing data/incomplete responses on the MQOL-HIV [12]	Discriminates between patients based on symptom severity, inpatient care and stage of illness [4]. MQOL-HIV scores distinguished between AIDS, symptomatic HIV, and asymptomatic cases, on 7 domains and overall QOL in one study however inadequate discrimination between disease stages was found in a Spanish study [20].	Good internal consistency (Cronbach’s alpha >0.70) for 8 of the 10 domains [4]. Poor internal consistency for physical and mental health, physical and sexual functioning [20]. Good test-retest reliability for all domains except cognitive functioning [4]. Poor test-retest reliability [20].	Somewhat responsive to change in the number of symptoms, viral load and CD4 count over a 3-month period [4]. Responsive to perceived changes in quality of life over 6 months in one study however only five dimensions were sensitive to clinical changes during ART [20]. Less sensitive than the MOS-HIV for detecting changes after starting or switching ART. MOS-HIV detected change on a greater number of subscales [12].	Floor and/or ceiling effects were reported in some dimensions [17]. Fewer problems with floor ceiling effects when compared to the MOS-20 [20].	The MQoL-HIV was not one of the reviewed measures recommended by Clayson et al. [4] A relatively sound and useful measure where attention has been paid to patient input and the breadth of the concept however the instrument is culture-specific [20].
PROQOL-HIV [38, 41]	8 scored domains: Physical health and symptoms, treatment impact, emotional distress, health concerns, body change, intimate relationships, social relationships, stigma and 4 additional items addressing religious beliefs, finance, having children and satisfaction with care.	7 mins [51] 43 items	Rated on a 5-point scale ranging from 0 = ‘never’ to 4 = “always”	not stated	not stated	not stated	not stated	not stated	No information reported in the reviews
MOS-HIV [36]	Two summary scores—the physical health score and mental health score and 10 domains: physical functioning, pain, social functioning, role functioning, emotional well-being, energy/fatigue, cognitive functioning, health distress, health transition, general health and overall quality of life	10 mins [4, 12] 35 items	2–6 response options per item	Translated into at least 14 languages, largely designed for industrialised world [20]. English version takes approx. 5–10 min [4, 12, 20] but twice as long has been reported for the use of some translations, e.g. Spanish, where more words are needed to express the concepts [20]. Scoring/ interpretation is complex [12, 20]. Less missing data than MQOL-HIV [12].	Mixed reports regarding construct validity with some suggesting poor construct validity [17] and others suggesting good construct validity [20]. Large body of evidence supporting convergent and discriminant validity [4, 12], although some studies have not found the expected relationship with CD4 count (construct validity) [12, 20].	Moderate /good internal consistency generally reported [4, 12, 19, 20] although Carabin reported good internal consistency for some but not all domains [17]. Inadequate test-retest reliability [17, 19].	Responsiveness has been established in a wide variety of contexts including adverse events, increased symptoms, opportunistic infections and AIDS-defining events, initiation of ART [4, 10, 12, 18]. Negligible effects in treatment experienced adults changing therapy [10]. Studies have found the MOS-HIV is more responsive than EQ-5D, HUI3 and MQOL-HIV [12].	Floor and ceiling effects have been reported [19, 20].	Well established reliability/validity and widely used but concerns that as one of the earliest HIV-specific measures to be developed it may not have continued relevance for PLWH. They question whether there is a true advantage to using the MOS-HIV over the SF-36. Would be unwise to administer alongside another MOS measure such as the SF-36 because of shared items [4]. May have limited value in cross-cultural research because although the scale has been translated into many languages, it may not have sematic and conceptual equivalence outside the USA [20]. More information is needed about performance of the scale in women, low income and other socially disadvantaged groups [20]. Can be administered individually or together with the EQ-5D to measure changes in HRQOL [10]. There is a lack of items addressing sexual function and body image [18]. Validation data draws on a range of patient groups from asymptomatic to those with advanced HIV [4].
WHOQOL-HIV BREF [39]	Physical, psychological, level of independence, social, environmental, and spiritual QoL	31 items	5 point likert scale	not stated	not stated	not stated	not stated	not stated	not stated
Symptom Quality of Life Adherence (HIV-SQUAD) [40]	HRQOL items include physical and psychological domains. The measure also includes symptoms and a visual analogue scale for adherence	24 items	5 point likert scales, dichotomous items and a visual analogue scale	not stated	The measure discriminated between patients at different CD4 counts and with/without hepatitis co-infection (construct validity) [12].	Cronbach’s alpha was acceptable for the physical component but <0.7 for the psychological component (internal consistency) [12]	Responsive to change in HIV viral load [12].	not stated	not stated

### *ACTG-SF21* [34]

The ACTG-21 consists of 21 items encompassing 7 HRQoL domains: physical functioning, energy/fatigue, social functioning, cognition, pain, health perception and emotional wellbeing. The reviews did not report reliability or validity data for the scale but floor and ceiling effects were anticipated given that the scale shares items with other MOS measures [17]. The measure has not been widely used in people with HIV. There was therefore insufficient information available to determine the suitability of this measure.

### HIV-QL-31 [35]

The HIV-QL-31 is a 31-item measure encompassing the following domains: sex life, pain, psychological aspects, relationships, limitations caused by HIV, symptoms and impact of treatment and care. The scale has high internal consistency [17, 20] and has been shown to discriminate between groups based on disease severity [20]. The measure was developed with patients and thereby covers a broad range of issues relevant to people with HIV [20]. However the HIV-QL-31 has not been widely used and the limited psychometric data available, including lack of information on responsiveness, make it difficult to establish the suitability of this measure.

### MOS-HIV [36]

The MOS-HIV is most widely used HIV-specific measure. Initially based on the SF-20, it consists of 35 items across 11 domains: physical functioning, pain, social functioning, role functioning, emotional well-being, energy/fatigue, cognitive function, health distress, health transition, general health and overall quality of life, allowing for the generation of physical and mental health summary scores. The instrument has been translated into at least 14 languages. The English version takes approximately 5–10 min to complete, but was reported to take twice as long for some translations, such as the Spanish version, where more words are required to express the concepts [20]. Scoring and interpretation has been described as complex [12, 20].

The reviews reported mixed findings on the psychometric properties of the MOS-HIV. Good internal consistency was generally reported [4, 12, 19, 20] however Carabin et al. reported acceptable internal consistency for some but not all domains [17]. Test-retest reliability was considered inadequate [17, 19]. The reviews contained mixed findings regarding construct validity, with some suggesting poor construct validity [17] and others suggesting good construct validity [20]. Acceptable convergent and discriminant validity was reported in several reviews [4, 12], however some studies did not find the expected relationship with CD4 count [12, 20]. Responsiveness has been established in a wide variety of contexts including adverse events, increased symptoms, opportunistic infections, AIDS defining events and initiation of ART [4, 10, 12, 18, 19], however negligible effects have been found in patients changing ART regimens [10]. Gakhar et al. found the MOS-HIV to be more responsive than the EQ-5D and HUI3 [12]. However, ceiling effects have been reported on several domains [19, 20].

Overall, the MOS-HIV was considered to have well-established psychometric properties. It was recommended as a suitable measure for assessing HRQoL in people with HIV [4, 10, 18], either individually or together with the EQ-5D [10]. However reservations expressed in the reviews included concerns about the continued relevance of the measure for people with HIV, given that it was one of the earliest HIV-specific scales to be developed [4], questions about whether there was a true advantage of using the MOS-HIV over the SF-36 [4] and scepticism about the ‘gold standard’ status that has been assigned to the MOS-HIV based on available evidence [20]. One criticism was that while patient interviews were conducted as part of the development process, they had not sampled extensively from people with HIV, limiting content validity [20]. Furthermore, like the SF-36, the MOS-HIV may lack conceptual relevance across different languages and cultures [20]. The need for more information about the performance of the scale in women, low income and disadvantaged groups was also identified [20].

### Multidimensional quality of life questionnaire for persons with HIV/AIDS (MQOL-HIV) [37]

The MQOL-HIV consists of 40 items assessing 10 domains: physical health, physical functioning, mental health, social functioning, cognitive functioning, social support, financial status, sexual functioning, partner intimacy and access to care. These domains were developed through interviews with HIV positive patients and providers in different settings.

The MQOL-HIV was considered to be a useful instrument since attention had been paid to the concerns of people living with HIV in its development [20], however there was limited evidence to support the construct validity, reliability and responsiveness of this instrument [4, 12, 20] and floor and/or ceiling effects were reported in some domains [17].

### PROQOL-HIV [38, 42]

The PROQOL-HIV consists of 43 items assessing 8 domains: physical health and symptoms, treatment impact, emotional distress, health concerns, body change, intimate relationships, social relationships and stigma. The items were developed through interviews conducted with people living with HIV in 9 countries. Psychometric data were not reported in the reviews.

### Symptom quality of life adherence (HIV-SQUAD) [40]

The HIV-SQUAD consists of 24 items assessing HRQoL, a symptom checklist and a visual analogue scale to measure adherence. Preliminary psychometric data were reported in one review [12]. While Cronbach’s alphas indicted acceptable internal consistency for the physical component, there was unacceptable internal consistency for the psychological component. The instrument was able to discriminate between patients with different illness states (CD4 count, hepatitis infection) and was responsive to changes in HIV viral load [12]. However, there was no evidence of the measure’s use beyond the initial validation paper and limited psychometric data were available in the reviews.

### WHOQOL-HIV BREF [39]

The WHOQOL-HIV BREF was developed in focus groups of people with HIV across 6 countries, ensuring that the items have conceptual and semantic relevance across cultures [20]. The instrument has 31 items, including several developed as a result of input from people with HIV (e.g. sexual activity, social inclusions and aspects of spirituality, such as forgiveness) and covers the six generic WHOQOL domains (physical functioning, psychological functioning, levels of independence, social relationships, environment and spirituality). No psychometric data on the scale were available in the reviews.

## Discussion

The aim of this review was to identify brief, appropriately validated, pragmatic tools for assessing HRQoL in HIV interventions and clinical care, by synthesising the findings of previous reviews on this topic. Both generic and HIV-Specific HRQoL instruments were identified.

Of the 10 generic measures, the EQ-5D, SF-36 and WHOQOL-BREF appear to be the most extensively used in HIV research. The reviews provided psychometric data to support the use of the EQ-5D and SF-36. A major advantage of the EQ-5D is its brevity, enabling most patients to complete the instrument approximately a minute. However, a limitation of the EQ-5D reported in the reviews was the potential ceiling effect. Indeed in one RCT, 40% patients obtained the highest possible score [43]. However, the papers included all reviewed studies using the original three level version of the EQ-5D, whereby patients had three response options for each item (no problems/ some or moderate problems/ extreme problems). A revised five level version is now available (no problems/ slight problems/ moderate problems/ severe problems/ extreme problems) which has demonstrated good psychometric qualities including acceptable internal consistency and a reduced ceiling effect among people with HIV [44].

The benefits of these generic HRQoL instruments include the availability of normative data allowing comparison of HRQOL with other groups, and the ability to examine the impact of HIV and comorbid conditions on HRQOL in a single assessment. Furthermore utility weights can be derived from both the EQ-5D and SF-36 enabling their use in economic analyses. However, generic measures may be less sensitive to changes in disease or treatment than HIV-specific instruments [11].

Of the HIV-specific measures, only the MOS-HIV was supported by sufficient psychometric data in the reviews, although there was some concern about limited input from people with HIV, cultural relevance and its continued applicability since the introduction of ART. Two more recently developed HIV-specific measures, the WHOQOL-HIV BREF and the PROQOL-HIV, were identified in the reviewed papers but their psychometric properties were not described.

A paper outlining the development and initial validation of the WHOQOL-HIV BREF was published in 2012 [39], reporting good internal consistency and discriminant validity. The authors highlighted the importance of the spirituality/personal beliefs and independence domains, often overlooked in quality of life research. They acknowledged the need for further validation, including in studies with longitudinal designs. Subsequent cross-sectional research has provided evidence of the measure’s psychometric properties in a range of populations including people with HIV in Portugal [45, 46], Taiwan [47], Malaysia [48], Vietnam [49] and Iran [50]. Preliminary evidence of its responsiveness comes from a prospective study of patients initiating ART in China, where significant improvements in QOL in all WHOQOL-HIV BREF domains were observed over the first six months of treatment [51].

The PROQOL-HIV was developed with extensive input from people living with HIV across nine countries [38]. It has been validated in a large cross-sectional multi-cultural study, demonstrating good internal consistency and construct validity [42]. In addition, satisfactory test-retest reliability has been established in a smaller sample [42]. An electronic version of the PROQOL-HIV has been found to be reliable and acceptable to patients [52]. Again, there is currently limited published research assessing the responsiveness of the PROQOL-HIV, although a recent trial reported a significant increase in scores on the body change, social relationships and emotional distress subscales following an online self-management intervention [53]. The PROQOL-HIV and WHOQOL-HIV BREF are increasingly being used in HIV research and may prove to be psychometrically sound and culturally valid alternatives to the MOS-HIV.

One strategy recommended within the reviews was to use a generic measure alongside an HIV-specific instrument [4, 10], such as the EQ-5D and the MOS-HIV [10]. Due to overlap in items it would not be appropriate to use the SF-36 alongside the MOS-HIV [4].

This review adds value to the literature by synthesising the findings of existing reviews of HRQoL measures used in HIV, including the findings of recent reviews on the subject [3, 10–12] which had various aims, for example to identify frequently used instruments [3]; determine how instruments have been applied to ART utilisation [12], identify measures used in a specific treatment context [11] and determine the responsiveness of specific instruments [10]. As with all reviews, we were limited by the data available in the included papers. The results of the quality assessment indicated that many of the reviews were of a poor quality, with most lacking a comprehensive search or identification/selection of papers by independent reviewers. No recent reviews with searches conducted since 2013 were identified. It is possible that new measures of quality of life could have been validated for use in people with HIV since this time. For example, a shorter version of the WHOQOL- BREF has subsequently been developed (EUROHIS-QOL-8) [54] but as yet there are insufficient validation data to recommend this measure.

For this review our aim was to identify brief measures for use in a busy clinic or repeated measures study. However, there may be HRQoL tools available that take longer than 10 min to complete or have more than 40 items but are still acceptable to patients and clinicians in the HIV care setting. For example the Functional Assessment of Human Immunodeficiency Virus Infection (FAHI [55]; 47 items, <15mins completion time [4, 56]) was viewed favourably in the review by Clayson and colleagues [4] and may be appropriate, although others have suggested it is too long and difficult to score for use in a clinical setting [57]. Further research is required to establish acceptability, which was rarely mentioned in the reviews. The selection of a HRQoL measure for use in research or clinical practice is also likely to be influenced by cost and the need to buy a licence. The reviews included in this review did not include information on the cost of the various instruments, therefore no licencing or cost information has been included in the current review.

The choice of one HRQoL measure over another is likely to be influenced by the purpose of the quality of life assessment and the domains of HRQoL that are most relevant to the specific research or clinical question. For example, responsiveness is essential where the aim is to evaluate treatment. Our review provided evidence of responsiveness for the EQ-5D, SF-36 and MOS-HIV. For clinical practice, it is important that a wider range of requirements are met, for example it is important that the scale is valid and reliable, but also simple to complete and score and easily interpretable [58]. The MOS-HIV and SF-36 include several different rating scales and response options, which could make scoring complex [20]. While psychometric data were lacking in the reviews, subsequent research suggests that the WHOQOL-HIV BREF and PROQOL-HIV may prove to be the most cross-culturally valid measures and therefore a good choice for international assessment of HRQOL in HIV.

## Conclusion

This systematic review of reviews identified several validated generic and HIV specific pragmatic tools for assessing HRQoL in HIV interventions and clinical care. The measures supported with most psychometric evidence in the systematic reviews were the EQ-5D, SF-36, WHOQOL-BREF and MOS-HIV. More recently developed HIV specific scales, including the PROQOL HIV and WHOQOL BREF-HIV may prove to be the most cross-culturally valid. Ultimately, the selection of a HRQoL measure is likely to be influenced by the context in which it is to be used.

## Abbreviations

ART: Antiretroviral therapy
NHS: National Health Service
NIHR: National Institute for Health Research
UK: United Kingdom
